# Desfechos em 2 Anos do Stent Inspiron™ com Polímero Biodegradável e Eluição de Sirolimo em um Cenário de Mundo Real

**DOI:** 10.36660/abc.20260244

**Published:** 2026-11-04

**Authors:** Ricardo Czarnobai Soccol, Henrique Barbosa Ribeiro, Ricardo Lasevitch, Denise Pellegrini, Expedito Eustáquio Ribeiro da Silva, Carlos Alberto Santos de Mattos, Augusto Lima, Noriaki Takeshita, Sílvio Sérgio Pontes Câmara, José de Ribamar Costa, Leandro Zacarias Figueiredo de Freitas, Marcelo Sabedotti, Pablo Tomé Teixeirense, Márcio Augusto Meirelles Truffa, Ricardo de Gasperi, Ewerton de Souza Abreu, Ismael Arcari Bassani, José Armando Mangione, Maurício Cavalieri, Gustavo Martins Pereira Alves, Pedro Beraldo de Andrade, Rafael Maestri, Rafael Rebêlo César Cavalcanti, Maria Sanali Paiva, Marcelo de Oliveira Hirga, José Airton de Arruda, Flávio Correa Pivatelli, Julia Gonçalves Zandomenico, Paulo R. A. Caramori

**Affiliations:** 1 Hospital São Lucas Pontifícia Universidade Católica do Rio Grande do Sul Porto Alegre RS Brasil Hospital São Lucas da Pontifícia Universidade Católica do Rio Grande do Sul, Porto Alegre, RS – Brasil; 2 Hospital das Clínicas Faculdade de Medicina Universidade de São Paulo São Paulo SP Brasil Instituto do Coração do Hospital das Clínicas da Faculdade de Medicina da Universidade de São Paulo, São Paulo, SP – Brasil; 3 Hospital de Clínicas de Passo Fundo Passo Fundo RS Brasil Hospital de Clínicas de Passo Fundo, Passo Fundo, RS – Brasil; 4 Santa Casa de Misericórdia de Belo Horizonte Belo Horizonte MG Brasil Santa Casa de Misericórdia de Belo Horizonte, Belo Horizonte, MG – Brasil; 5 Hospital Angelina Caron Campina Grande do Sul PR Brasil Hospital Angelina Caron, Campina Grande do Sul, PR – Brasil; 6 Hospital e Maternidade São Marcos Teresina PI Brasil Hospital e Maternidade São Marcos, Teresina, PI – Brasil; 7 Instituto Dante Pazzanese de Cardiologia São Paulo SP Brasil Instituto Dante Pazzanese de Cardiologia, São Paulo, SP – Brasil; 8 Santa Casa de Misericórdia de Goiânia Goiânia GO Brasil Santa Casa de Misericórdia de Goiânia, Goiânia, GO – Brasil; 9 Universidade de Caxias do Sul Caxias do Sul RS Brasil Universidade de Caxias do Sul, Caxias do Sul, RS – Brasil; 10 Hospital dos Fornecedores de Cana de Piracicaba Piracicaba SP Brasil Hospital dos Fornecedores de Cana de Piracicaba, Piracicaba, SP – Brasil; 11 Hospital Unimed Piracicaba Piracicaba SP Brasil Hospital Unimed Piracicaba, Piracicaba, SP – Brasil; 12 Hospital Paulo Sacramento Jundiaí SP Brasil Hospital Paulo Sacramento, Jundiaí, SP – Brasil; 13 Associação Dr. Bartholomeu Tacchini Hospital Tacchini Bento Goncalves RS Brasil Associação Dr. Bartholomeu Tacchini (Hospital Tacchini), Bento Goncalves, RS – Brasil; 14 Hospital PRONTOCOR de Muriaé Muriaé MG Brasil Hospital PRONTOCOR de Muriaé, Muriaé, MG – Brasil; 15 Hospital Beneficência Portuguesa de Pelotas Pelotas RS Brasil Hospital Beneficência Portuguesa de Pelotas, Pelotas, RS – Brasil; 16 Beneficência Portuguesa de São Paulo São Paulo SP Brasil Beneficência Portuguesa de São Paulo, São Paulo, SP – Brasil; 17 Hospital Alemão Oswaldo Cruz São Paulo SP Brasil Hospital Alemão Oswaldo Cruz, São Paulo, SP – Brasil; 18 Hospital São Francisco de Assis Belo Horizonte MG Brasil Hospital São Francisco de Assis, Belo Horizonte, MG – Brasil; 19 Samer Hospital Resende RJ Brasil Samer Hospital, Resende, RJ – Brasil; 20 Santa Casa de Misericórdia de Marília Marília SP Brasil Santa Casa de Misericórdia de Marília, Marília, SP – Brasil; 21 Unimed Litoral Balneário Camboriú SC Brasil Unimed Litoral, Balneário Camboriú, SC – Brasil; 22 Maestri e Kormann BC Balneário Camboriú SC Brasil Maestri e Kormann BC, Balneário Camboriú, SC – Brasil; 23 Hospital Memorial Arthur Ramos Maceió AL Brasil Hospital Memorial Arthur Ramos, Maceió, AL – Brasil; 24 Rede D’Or Maceió AL Brasil Rede D’Or, Maceió, AL – Brasil; 25 Instituto Atena de Pesquisa Clínica Maceió AL Brasil Instituto Atena de Pesquisa Clínica, Maceió, AL – Brasil; 26 Hospital Policlin São José dos Campos SP Brasil Hospital Policlin, São José dos Campos, SP – Brasil; 27 Hospital Meridiona Vitória ES Brasil Hospital Meridional, Vitória, ES – Brasil; 28 Hospital de Base São José do Rio Preto SP Brasil Hospital de Base, São José do Rio Preto, SP – Brasil

**Keywords:** Polímeros Biodegradáveis, Intervenção Coronária Percutânea, Stents farmacológicos, Sirolimo

## Abstract

**Fundamento:**

O Inspiron™ é um stent coronário com eluição de sirolimo (SES) de terceira geração, com hastes finas e revestimento abluminal de polímero biodegradável. Embora amplamente utilizado no Brasil, ainda são limitadas as evidências de longo prazo provenientes de estudos em condições de mundo real.

**Objetivos:**

Avaliar a segurança e a efetividade do SES Inspiron™ ao longo de 24 meses em uma população não selecionada submetida à intervenção coronária percutânea (ICP).

**Métodos:**

Esta análise agrupada incluiu 2.803 pacientes consecutivos submetidos ao implante do SES Inspiron™ entre junho de 2017 e janeiro de 2022 em 25 centros brasileiros. O desfecho primário foi a ocorrência de eventos cardiovasculares adversos maiores (ECAM), definidos como o composto de morte cardiovascular, infarto agudo do miocárdio (IAM) e revascularização da lesão-alvo (RLA) em 24 meses. Foram utilizadas análise de sobrevida de Kaplan-Meier, regressão de riscos proporcionais de Cox e modelos com *splines* cúbicas restritas. Valores de p bicaudais < 0,05 foram considerados estatisticamente significativos.

**Resultados:**

Aos 24 meses, a incidência cumulativa de ECAM foi de 6,7%, com taxas de 5,4% para mortalidade por todas as causas, 2,3% para mortalidade cardiovascular, 1,2% para IAM, 0,9% para RLA e 0,6% para trombose do *stent* (TS) definitiva ou provável. Pacientes com diâmetro do vaso < 2,5 mm apresentaram taxas significativamente mais elevadas de ECAM, morte cardiovascular, nova revascularização e TS (p = 0,022). Os preditores independentes de ECAM incluíram idade mais avançada (p = 0,034), doença do tronco da coronária esquerda ou doença coronariana triarterial (p = 0,024) e síndrome coronariana aguda como indicação para ICP (p < 0,001).

**Conclusões:**

Neste grande registro de mundo real, o SES Inspiron™ demonstrou perfil favorável de segurança e efetividade ao longo de 24 meses, com baixas taxas de TS e nova revascularização, apesar da inclusão de lesões coronárias complexas.

## Graphical abstract

**Figura Central:**
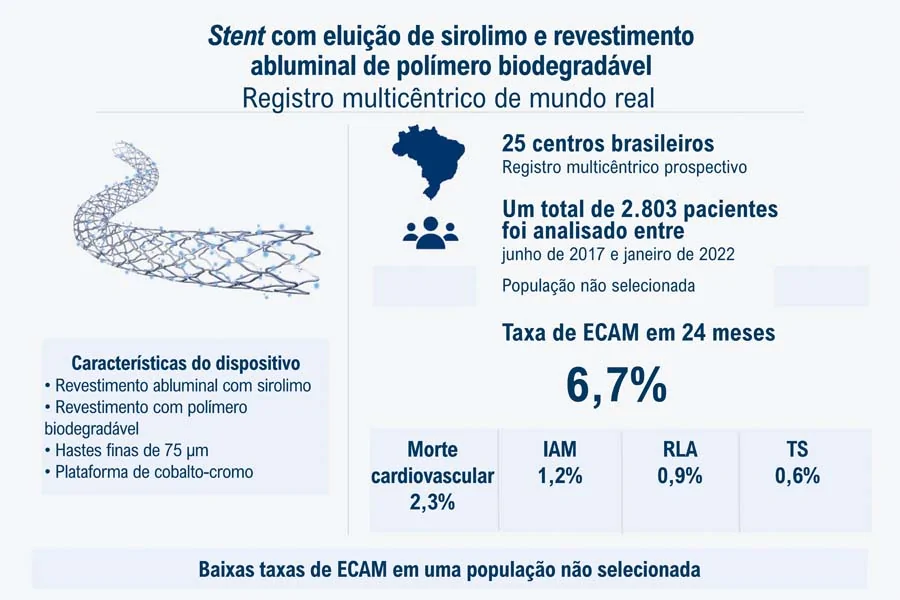
Desfechos em 2 Anos do Stent Inspiron™ com Polímero Biodegradável e Eluição de Sirolimo em um Cenário de Mundo Real

ECAM: eventos cardiovasculares adversos maiores; IAM: infarto agudo do miocárdio; RLA: revascularização da lesão-alvo; TS: trombose do stent.

## Introdução

A intervenção coronária percutânea (ICP) está entre os procedimentos terapêuticos mais frequentemente realizados na prática cardiovascular contemporânea.^1^ A introdução dos *stents* farmacológicos (SF) reduziu de forma significativa a reestenose intra-*stent* ao permitir a liberação local de agentes antiproliferativos na parede arterial.^1,2^ Entretanto, os SF de primeira geração foram associados a maior trombogenicidade e a um risco mais elevado de trombose do *stent* (TS) tardia e muito tardia.^3,4^ Revestimentos poliméricos permanentes e plataformas de *stents* com hastes espessas têm sido implicados no retardo da cicatrização vascular, na inflamação persistente e no aumento da trombogenicidade.^5,6^ Essas limitações impulsionaram o desenvolvimento de SF de nova geração com hastes mais finas e revestimentos de polímero biodegradável.^7,8^ Quando aplicados exclusivamente à superfície abluminal do *stent*, os polímeros biodegradáveis podem favorecer ainda mais a cicatrização vascular, reduzindo simultaneamente o risco de eventos cardiovasculares adversos maiores (ECAM) tardios.^3^

O *stent* com eluição de sirolimo (SES) Inspiron™ (Scitech Medical™, Aparecida de Goiânia, Brasil) foi desenvolvido com base nesses princípios. No estudo INSPIRON-I, o dispositivo apresentou perda luminal tardia e obstrução neointimal significativamente menores do que com o stent não farmacológico correspondente.^9^ Em um ensaio clínico randomizado (ECR) comparando o Inspiron™ com um *stent* com eluição de biolimus, não foram observadas diferenças angiográficas significativas aos 9 meses, e os desfechos clínicos permaneceram comparáveis ao longo de 5 anos de seguimento.^10,11^ Estudos de imagem intravascular também demonstraram cicatrização vascular favorável e altas taxas de cobertura das hastes do *stent* após o implante do Inspiron™.^12,13^

Após a obtenção da marca CE (Conformité Européenne) em 2020 e sua subsequente ampla adoção na prática clínica, evidências de mundo real tornaram-se essenciais para avaliar a segurança e a efetividade do dispositivo em longo prazo. Em particular, ainda são escassos os dados contemporâneos em populações consecutivas, de alto risco e com anatomia coronária complexa, tratadas na prática clínica de rotina. Portanto, este estudo teve como objetivo avaliar os desfechos clínicos de longo prazo em condições de mundo real associados ao SES Inspiron™.

## Métodos

### Delineamento do estudo e população

Os estudos INSPIRON (identificador no ClinicalTrials.gov: NCT03263260) e INSPIRON LATITUDE (identificador no ClinicalTrials.gov: NCT03471234) foram registros prospectivos multicêntricos conduzidos em 25 centros brasileiros. Devido ao delineamento semelhante dos estudos, os dados individuais dos pacientes de ambos os registros foram agrupados para a presente análise. Entre junho de 2017 e janeiro de 2022, foram incluídos 2.803 pacientes consecutivos submetidos à ICP para tratamento de lesão coronária nativa com implante do *stent* Inspiron™.

Foram elegíveis pacientes com idade igual ou superior a 18 anos, apresentando sintomas de angina e/ou isquemia miocárdica documentada, submetidos ao tratamento de uma lesão coronária nativa com o SES. Os critérios angiográficos de elegibilidade incluíram comprimento da lesão < 34 mm e diâmetro de referência do vaso de 2,5-4,0 mm. Os critérios de exclusão compreenderam lesões-alvo localizadas em enxertos de veia safena ou enxertos arteriais e o tratamento com qualquer *stent* diferente do dispositivo do estudo durante o procedimento índice.

Todos os procedimentos foram realizados de acordo com a prática clínica habitual. A escolha do acesso vascular, do tamanho do *stent* e da estratégia intervencionista ficou a critério do operador responsável. O diâmetro médio do vaso foi definido como a média dos diâmetros de referência dos vasos correspondentes às lesões tratadas durante o procedimento índice e foi determinado de acordo com a avaliação angiográfica do operador durante a ICP. A angiografia coronária quantitativa foi incentivada sempre que viável; entretanto, a estimativa angiográfica visual foi utilizada na maioria dos casos, refletindo a prática clínica rotineira em condições de mundo real. O uso de imagem intracoronária não foi exigido pelo protocolo do estudo. Foi recomendada terapia antiplaquetária dupla com aspirina e um inibidor de P2Y12 por pelo menos 6 meses após o implante do *stent*.

O estudo foi conduzido de acordo com a Declaração de Helsinque e aprovado pelo comitê de ética em pesquisa envolvendo seres humanos de cada centro participante. Todos os pacientes forneceram consentimento informado por escrito antes da inclusão.

### Desenho do *stent*

O dispositivo do estudo é um SES de cobalto-cromo com hastes finas (75 μm) e revestimento abluminal de polímero biodegradável composto por poli-L-ácido lático e poli(ácido lático-co-glicólico). O dispositivo libera sirolimo em baixa dose (1,4 µg/mm^2^), resultando em um perfil moderado de liberação do fármaco, com 60% do fármaco liberado em até 10 dias e liberação completa em 45 dias. A degradação completa do polímero ocorre entre 6 e 9 meses (dados em arquivo, Scitech Medical™, Brasil). O estudo REPAIR avaliou o perfil de cicatrização vascular do *stent* Inspiron™ por meio de tomografia de coerência óptica (TCO) seriada e demonstrou elevada taxa de cobertura das hastes, com 97% das hastes cobertas aos 3 meses.^13^

### Desfechos do estudo

O desfecho primário foi a incidência de ECAM, definido como o desfecho composto de morte cardiovascular, infarto agudo do miocárdio (IAM) e revascularização da lesão-alvo (RLA) em 24 meses. Os desfechos secundários incluíram mortalidade por todas as causas, mortalidade cardiovascular, qualquer IAM, RLA, revascularização do vaso-alvo (RVA) e trombose definitiva ou provável do *stent* (TS) em 24 meses.

### Definições

O sucesso do dispositivo foi definido como a entrega e implantação bem-sucedidas do *stent* do estudo, com estenose residual < 30%, avaliada por angiografia coronária quantitativa ou estimativa visual. O sucesso do procedimento foi definido como sucesso do dispositivo sem ocorrência de óbito hospitalar, IAM com supradesnivelamento do segmento ST, TS aguda ou subaguda, ou necessidade de RVA de emergência.

O IAM foi definido de acordo com a Terceira Definição Universal contemporânea.^14^ Em conformidade com os critérios do Academic Research Consortium,^15^ todos os óbitos foram classificados como cardíacos, exceto quando uma causa não cardíaca inequívoca fosse estabelecida. A TS definitiva foi definida como trombose confirmada angiograficamente com fluxo Thrombolysis in Myocardial Infarction grau 0 ou 1, ou pela presença de trombo com limitação de fluxo associado a sintomas isquêmicos agudos, independentemente da ocorrência de qualquer procedimento de revascularização no intervalo. A TS foi classificada quanto ao momento de ocorrência em aguda (0-24 horas), subaguda (> 24 horas a 30 dias), tardia (30 dias a 1 ano) ou muito tardia (> 1 ano). A TS provável foi definida como morte inexplicada em até 30 dias ou IAM do vaso-alvo sem confirmação angiográfica de trombose ou identificação de outra lesão culpada. A TS possível foi definida como morte inexplicada ocorrida após 30 dias.

A RLA foi definida como qualquer ICP repetida da lesão-alvo realizada para tratamento de reestenose. A lesão-alvo compreendeu o segmento tratado estendendo-se 5 mm proximal e 5 mm distalmente às bordas do *stent*. A RVA foi definida como qualquer ICP repetida envolvendo qualquer segmento do vaso-alvo.

### Seguimento clínico

Os dados clínicos, angiográficos e procedimentais basais foram coletados prospectivamente em um banco de dados específico em cada centro participante. Todos os pacientes foram acompanhados até a alta hospitalar. O seguimento clínico foi programado para 30 dias, 12 meses e 24 meses após o procedimento índice e realizado por meio de consultas ambulatoriais ou entrevistas telefônicas estruturadas, de acordo com a prática institucional local. Informações adicionais foram obtidas por meio da revisão de prontuários médicos e de comunicação direta com os médicos assistentes sempre que necessário. Os eventos clínicos foram reportados por cada centro participante de acordo com as definições previamente especificadas no protocolo. O centro coordenador do estudo realizou revisão adicional dos eventos clínicos reportados e dos dados de seguimento, quando necessário, para garantir a completude, a precisão e a consistência da adjudicação dos eventos clínicos entre os centros participantes.

### Análise estatística

As análises estatísticas foram realizadas utilizando o banco de dados agrupado dos registros INSPIRON e INSPIRON LATITUDE, composto por 2.803 pacientes submetidos à ICP. Os pacientes foram categorizados de acordo com os tercis do diâmetro médio do vaso (< 2,5 mm, 2,5-3,0 mm e > 3,0 mm) e do comprimento total dos *stents* implantados durante o procedimento índice (< 25 mm, 25-30 mm e > 30 mm).

O desfecho primário foi ECAM, definido como o desfecho composto de morte cardiovascular, IAM e RLA. Os desfechos secundários incluíram: i) o desfecho composto de mortalidade por todas as causas, IAM e RLA; ii) mortalidade por todas as causas; iii) mortalidade cardiovascular; iv) qualquer IAM; v) IAM do vaso-alvo; vi) RLA; vii) RVA; e viii) TS definitiva ou provável.

As variáveis contínuas apresentaram distribuição normal e foram expressas como média ± desvio-padrão. As comparações entre os três grupos foram realizadas por análise de variância de uma via (ANOVA), seguida do teste post hoc de Tukey para comparações múltiplas, quando apropriado. As variáveis categóricas foram comparadas pelo teste do qui-quadrado.

A sobrevida livre de eventos foi estimada pelo método de Kaplan-Meier e comparada pelo teste de *log-rank*. Modelos de regressão de riscos proporcionais de Cox univariados e multivariados foram utilizados para avaliar as associações do diâmetro médio do vaso e do comprimento total do *stent* com a ocorrência de ECAM durante o seguimento de 24 meses. A suposição de riscos proporcionais foi avaliada por meio dos resíduos de Schoenfeld, não sendo detectadas violações significativas.

Para avaliar possíveis associações não lineares entre o diâmetro médio do vaso ou o comprimento total do *stent* e a probabilidade de ECAM, foram construídos modelos de regressão com *splines* cúbicas restritas com cinco nós, utilizando a função *rcspline.plot* do pacote *Hmisc* no R. Foram geradas estimativas paramétricas e não paramétricas da função de regressão logística utilizando o algoritmo de suavização SuperSmoother de Friedman (*supsmu*).

Todos os testes estatísticos foram bicaudais, sendo considerado estatisticamente significativo um valor de p < 0,05. As análises estatísticas foram realizadas no R versão 3.5.3 (R Foundation for Statistical Computing) e no RStudio versão 1.1.4.

## Resultados

Um total de 2.803 pacientes foi incluído na presente análise. As características clínicas basais de acordo com o diâmetro médio do vaso estão resumidas na Tabela 1. Os pacientes com menor diâmetro do vaso eram mais idosos e havia maior proporção de mulheres. O diabetes melito (DM), independentemente do uso de insulina, foi mais prevalente entre os pacientes com diâmetro do vaso < 2,5 mm.. Dislipidemia e ICP prévia também foram mais frequentes nesse grupo. Em contrapartida, o tabagismo atual foi mais frequente entre os pacientes com diâmetro do vaso > 3,0 mm. Notavelmente, mais da metade da população do estudo apresentou síndrome coronariana aguda (SCA), ressaltando o caráter de mundo real e de alto risco da coorte.

**Tabela 1:** – Características clínicas basais de acordo com o diâmetro médio do vaso

Variável	Geral (n = 2.803)	Q1: < 2,5 mm (n = 688)	Q2: 2,5-3,0 mm (n = 1.263)	Q3: > 3,0 mm (n = 852)	Valor de p
Idade, anos	62,89 ± 10,81	64,14 ± 10,58	62,81 ± 10,56	62,00 ± 11,27	0,001
Sexo masculino, n (%)	1.812 (64,6)	383 (55,7)	811 (64,2)	618 (72,5)	< 0,001
HAS, n (%)	2.133 (76,1)	533 (77,5)	971 (76,9)	629 (73,8)	0,169
DM, n (%)	1.024 (36,5)	312 (45,3)	472 (37,4)	240 (28,2)	< 0,001
Dislipidemia, n (%)	1.459 (52,1)	390 (56,7)	645 (51,1)	424 (49,8)	0,017
Ex-tabagismo, n (%)	645 (23,0)	148 (21,5)	280 (22,2)	217 (25,5)	0,117
Tabagismo atual, n (%)	557 (19,9)	112 (16,3)	243 (19,2)	202 (23,7)	0,001
IAM prévio, n (%)	16 (0,6)	4 (0,6)	8 (0,6)	4 (0,5)	0,869
DRC, n (%)	97 (3,5)	19 (2,8)	48 (3,8)	30 (3,5)	0,484
CRM prévia, n (%)	112 (4,0)	33 (4,8)	50 (4,0)	29 (3,4)	0,380
ICP prévia, n (%)	493 (17,6)	144 (20,9)	207 (16,4)	142 (16,7)	0,029
Apresentação clínica					0,011
Angina estável, n (%)	1.369 (48,8)	367 (53,5)	585 (49,4)	379 (44,8)	
SCA, n (%)	1.434 (51,1)	321 (46,5)	640 (50,6)	473 (55,4)	

CRM: cirurgia de revascularização miocárdica; DM: diabetes melito; DRC: doença renal crônica; HAS: hipertensão arterial sistêmica; IAM: infarto agudo do miocárdio; ICP: intervenção coronária percutânea; SCA: síndrome coronariana aguda.

As características basais de acordo com o comprimento total do *stent* são apresentadas na Tabela 2. Os pacientes tratados com *stents* mais longos apresentaram maior probabilidade de serem do sexo masculino e de apresentar SCA na admissão. O tabagismo atual também foi mais prevalente entre os pacientes que receberam *stents* mais longos.

**Tabela 2:** – Características angiográficas e procedimentais de acordo com o comprimento total do *stent*

Variável	Geral (n = 2.803)	< 25 mm (n = 1.944)	25-30 mm (n = 419)	> 30 mm (n = 440)	Valor de p
**Vaso coronário-alvo**					
DA, n (%)	1.187 (42,3)	842 (43,3)	176 (42,0)	169 (38,4)	0,178
Cx, n (%)	512 (18,3)	384 (19,8)	63 (15,0)	65 (14,8)	0,018
CD, n (%)	1.038 (37,0)	685 (35,2)	169 (40,3)	184 (41,8)	0,021
Tronco da coronária esquerda, n (%)	34 (1,2)	24 (1,2)	1 (0,2)	9 (2,0)	0,053
**Tipo de lesão**					
Lesão *de novo*, n (%)	2.638 (94,1)	1.834 (94,3)	391 (93,3)	413 (93,9)	0,721
Reestenose intra-*stent*, n (%)	165 (5,9)	110 (5,7)	28 (6,7)	27 (6,1)	0,721
Diâmetro médio do vaso, mm	3,00 ± 0,45	2,98 ± 0,47	3,00 ± 0,44	3,04 ± 0,35	0,038
Comprimento médio da lesão, mm	21,02 ± 10,41	16,24 ± 5,57	27,44 ± 7,36	35,98 ± 11,72	< 0,001
Estenose pré-dilatação, %	85,93 ± 10,20	85,40 ± 10,06	86,51 ± 10,41	87,75 ± 10,39	< 0,001
Estenose pós-dilatação, %	1,75 ± 4,36	1,62 ± 4,12	1,83 ± 4,51	2,21 ± 5,03	0,084
Número de *stents* implantados por ICP	1,38 ± 0,96	1,37 ± 0,96	1,51 ± 1,04	1,32 ± 0,90	0,008
Comprimento médio do *stent*, mm	22,81 ± 8,18	18,33 ± 3,57	28,36 ± 1,17	37,28 ± 6,42	< 0,001
Diâmetro médio do *stent*, mm	3,00 ± 0,45	2,98 ± 0,47	3,00 ± 0,44	3,04 ± 0,35	0,038

CD: artéria coronária direita; Cx: artéria circunflexa esquerda; DA: artéria descendente anterior; ICP: intervenção coronária percutânea.

O comprimento da lesão aumentou progressivamente com o aumento do diâmetro do vaso. Os pacientes com vasos menores necessitaram de um maior número de *stents*, apesar de apresentarem menor comprimento médio dos *stents*. O diâmetro médio do *stent* aumentou proporcionalmente entre as categorias de diâmetro do vaso. As taxas de sucesso do procedimento foram uniformemente elevadas (> 99%) e não diferiram significativamente entre os grupos.

Quando os pacientes foram estratificados de acordo com o comprimento total do *stent*, a complexidade da lesão aumentou progressivamente entre as categorias. Os pacientes que receberam *stents* mais longos apresentaram lesões significativamente mais extensas e maior gravidade da estenose basal.

Aos 24 meses, a incidência do desfecho primário, ECAM, diferiu significativamente entre os grupos de diâmetro do vaso (p = 0,022). Os pacientes com diâmetro do vaso < 2,5 mm apresentaram a maior incidência de ECAM (5,4%), enquanto aqueles com diâmetro do vaso > 3,0 mm apresentaram a menor incidência (2,7%). A mortalidade cardiovascular também diferiu significativamente entre os grupos, ocorrendo com maior frequência nos pacientes com vasos menores (3,6% vs. 2,1% vs. 1,4%; p = 0,013).

Embora as taxas cumulativas brutas de eventos em 24 meses tenham diferido significativamente entre os grupos de diâmetro do vaso, a análise de Kaplan-Meier não demonstrou diferenças significativas na sobrevida livre de eventos pelo teste de *log-rank* (p = 0,37). Essa discrepância pode refletir diferenças no momento de ocorrência dos eventos, nos padrões de censura e no número relativamente pequeno de ECAM durante o seguimento. A análise de Kaplan-Meier mostrou aumento precoce de ECAM entre os pacientes com diâmetro do vaso < 2,5 mm durante os primeiros 60 dias após a ICP, seguido por novo aumento após aproximadamente 16 meses de seguimento (Figura 1A). Os pacientes com diâmetro intermediário do vaso (2,5-3,0 mm) apresentaram aumento tardio das taxas de eventos, iniciando-se aproximadamente aos 12 meses.

**Figura 1:**
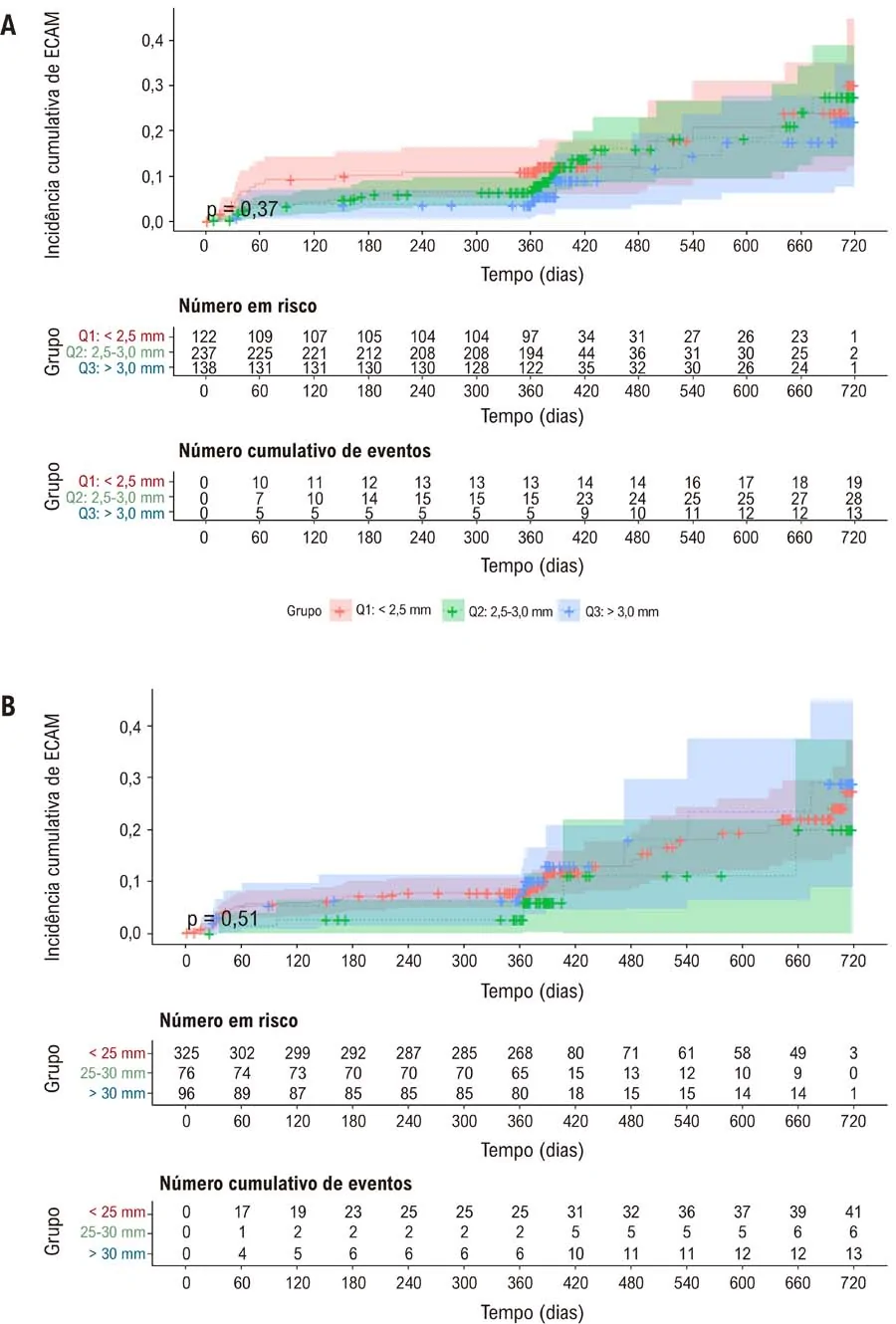
– Curvas de Kaplan-Meier para ECAM aos 24 meses de seguimento. A) Estratificadas de acordo com o diâmetro médio do vaso. B) Estratificadas de acordo com o comprimento total do stent (soma dos comprimentos de todos os stents implantados durante a intervenção coronária percutânea). ECAM: eventos cardiovasculares adversos maiores.

As taxas de RLA e TS também foram significativamente maiores entre os pacientes com menor diâmetro do vaso. Em contraste, não foram observadas diferenças significativas na mortalidade por todas as causas ou no IAM.

A análise por *splines* cúbicas restritas demonstrou associação global significativa entre o diâmetro médio do vaso e a probabilidade de ECAM, sugerindo uma relação não linear entre o calibre do vaso e ECAM (Figura 2). A curva de *spline* indicou redução progressiva do risco estimado de ECAM à medida que o diâmetro do vaso aumentava.

**Figura 2:**
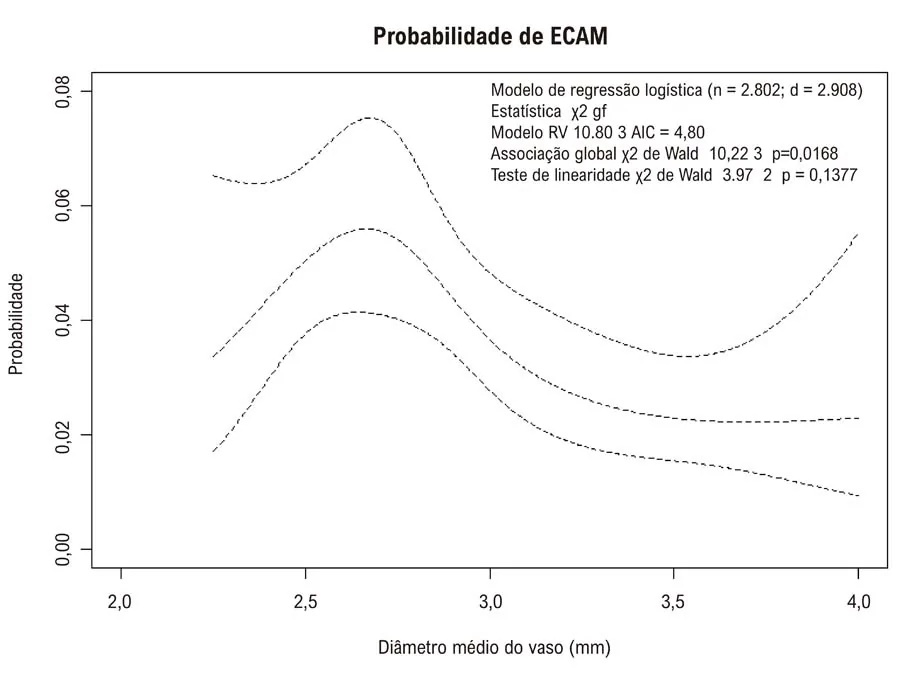
– Regressão por splines cúbicas restritas da probabilidade de ECAM (morte cardiovascular, infarto agudo do miocárdio ou revascularização da lesão-alvo) de acordo com o diâmetro médio do vaso. AIC: critério de informação de Akaike; ECAM: eventos cardiovasculares adversos maiores; gl: graus de liberdade; RV: razão de verossimilhança.

Na análise de regressão de Cox univariada, o diâmetro médio do vaso esteve significativamente associado a ECAM (p = 0,022). Entretanto, após ajuste multivariado por regressão de Cox com seleção stepwise, o diâmetro médio do vaso deixou de ser um preditor independente de ECAM. O modelo multivariado final identificou idade mais avançada (p = 0,034), doença do tronco da coronária esquerda ou doença arterial coronariana triarterial (p = 0,024) e SCA como indicação para ICP (p < 0,001) como preditores independentes de ECAM, com índice de concordância de 0,71. Esses achados são apresentados na Figura 3.

**Figura 3:**
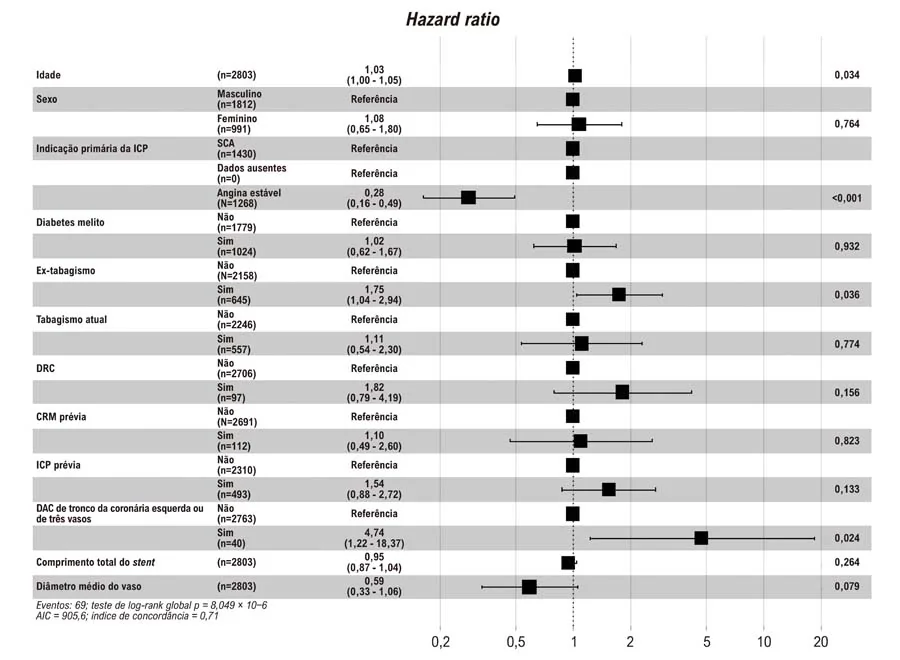
– Modelo de regressão de Cox multivariada com seleção stepwise para eventos cardiovasculares adversos maiores (morte cardiovascular, infarto agudo do miocárdio e revascularização da lesão-alvo) durante o seguimento de 24 meses. AIC: critério de informação de Akaike; CRM: cirurgia de revascularização miocárdica; DAC: doença arterial coronariana; DRC: doença renal crônica; ICP: intervenção coronária percutânea; SCA: síndrome coronariana aguda.

A incidência de ECAM aumentou numericamente com o aumento do comprimento total do *stent*, de 3,5% entre os pacientes tratados com stents < 25 mm para 5,7% entre aqueles que receberam *stents* > 30 mm; entretanto, essa diferença não atingiu significância estatística (p = 0,070).

Da mesma forma, as curvas de Kaplan-Meier demonstraram curvas de incidência cumulativa de ECAM semelhantes entre as categorias de comprimento do *stent* ao longo dos 24 meses de seguimento, sem diferenças significativas pelo teste de *log-rank* (p = 0,51) (Figura 1B).

Entretanto, o maior comprimento total do *stent* esteve significativamente associado ao desfecho secundário composto de mortalidade por todas as causas, IAM ou RLA (p = 0,018). Esse achado pareceu ser impulsionado principalmente por uma taxa numericamente maior de mortalidade por todas as causas entre os pacientes que receberam *stents* > 30 mm aos 24 meses (7,3%; p = 0,100).

A análise por *splines* cúbicas restritas sugeriu aumento gradual da probabilidade estimada de ECAM com o aumento do comprimento total do *stent*, embora sem evidências de uma associação linear forte (Figura 4). Na análise de regressão de Cox multivariada, o comprimento total do *stent* não esteve independentemente associado a ECAM (p = 0,264).

**Figura 4:**
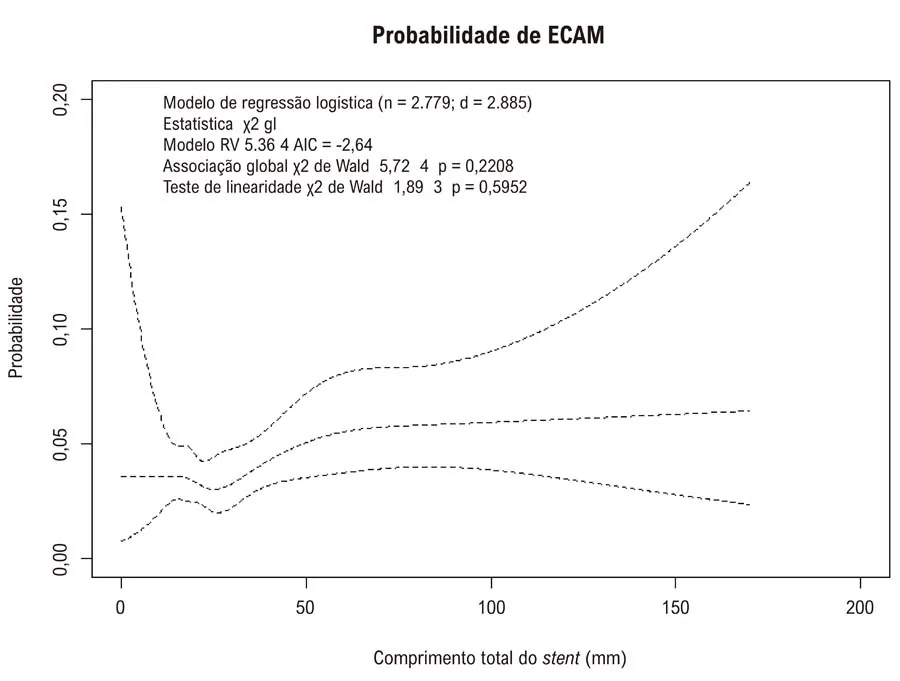
– Regressão por splines cúbicas restritas da probabilidade de ECAM (morte cardiovascular, infarto agudo do miocárdio ou revascularização da lesão-alvo) de acordo com o comprimento total do stent (soma dos comprimentos de todos os stents implantados durante a intervenção coronária percutânea). AIC: critério de informação de Akaike; ECAM: eventos cardiovasculares adversos maiores; gl: graus de liberdade; RV: razão de verossimilhança.

Os pacientes que desenvolveram ECAM durante o seguimento apresentaram maior probabilidade de ter SCA, doença renal crônica, CRM prévia, ICP prévia e histórico de tabagismo. Além disso, apresentaram comprimento total do *stent* significativamente maior e menor diâmetro médio do *stent* em comparação aos pacientes que não desenvolveram ECAM (Tabela 3).

**Tabela 3:** – Características clínicas e procedimentais de acordo com a ocorrência de ECAM durante o seguimento de 24 meses

Variável	Sem ECAM (n = 2.697)	ECAM (n = 106)	Valor de p
Idade, anos	62,74 ± 10,79	66,12 ± 10,85	0,002
Sexo masculino, n (%)	1.748 (64,8)	64 (60,4)	0,366
SCA, n (%)	1.367 (50,7)	67 (63,2)	0,014
HAS, n (%)	2.048 (75,9)	85 (80,2)	0,318
DM, n (%)	973 (36,1)	51 (48,1)	0,015
Dislipidemia, n (%)	1.397 (51,8)	62 (58,5)	0,182
Tabagismo atual, n (%)	530 (19,7)	27 (25,5)	0,159
Ex-tabagismo, n (%)	604 (22,4)	41 (38,7)	< 0,001
DRC, n (%)	86 (3,2)	11 (10,4)	< 0,001
IAM prévio, n (%)	14 (0,5)	2 (1,9)	0,082
CRM prévia, n (%)	101 (3,7)	11 (10,4)	0,001
ICP prévia, n (%)	456 (16,9)	37 (34,9)	< 0,001
DAC de tronco da coronária esquerda ou de três vasos, n (%)	278 (10,3)	23 (21,7)	< 0,001
Diâmetro médio do vaso, mm	3,01 ± 0,45	2,82 ± 0,41	< 0,001
Comprimento médio da lesão, mm	20,79 ± 10,28	26,11 ± 12,48	< 0,001
Comprimento total do *stent*, mm	30,92 ± 24,91	47,30 ± 36,42	< 0,001
Número de *stents* implantados	1,36 ± 0,94	1,74 ± 1,18	< 0,001
Diâmetro médio do *stent*, mm	3,01 ± 0,45	2,78 ± 0,39	< 0,001
Comprimento médio do *stent*, mm	22,63 ± 8,07	27,42 ± 9,84	< 0,001
Sucesso angiográfico, n (%)	2,498 (99,8)	99 (99,0)	0,089

CRM: cirurgia de revascularização miocárdica; DAC: doença arterial coronariana; DM: diabetes melito; DRC: doença renal crônica; ECAM: eventos cardiovasculares adversos maiores; HAS: hipertensão arterial sistêmica; IAM: infarto agudo do miocárdio; ICP: intervenção coronária percutânea; SCA: síndrome coronariana aguda.

Em conjunto, esses achados sugerem que o menor diâmetro dos vasos coronários está associado a desfechos cardiovasculares menos favoráveis após ICP, particularmente em relação à mortalidade cardiovascular e à necessidade de nova revascularização, enquanto o comprimento total do *stent* parece representar um marcador da complexidade da lesão, e não um preditor independente de ECAM após ajuste multivariado.

Os principais desfechos clínicos em 12 e 24 meses são apresentados na Tabela 4. A incidência cumulativa de ECAM aumentou progressivamente ao longo do tempo, acompanhada por aumentos graduais na mortalidade por todas as causas, mortalidade cardiovascular, IAM, nova revascularização e TS. De modo geral, as taxas de ECAM permaneceram baixas durante todo o período do estudo. Esses achados também estão resumidos na Figura Central.

**Tabela 4:** – Desfechos clínicos aos 12 e 24 meses de seguimento

Desfecho	12 meses (n = 2.803)	24 meses (n = 2.803)
ECAM, n (%)	106 (3,8)	189 (6,7)
Mortalidade por todas as causas, n (%)	101 (3,6)	151 (5,4)
Mortalidade cardiovascular, n (%)	46 (1,6)	64 (2,3)
IAM, n (%)	27 (1,0)	35 (1,2)
RVA, n (%)	Não reportado	33 (1,2)
RLA, n (%)	Não reportado	26 (0,9)
TS definitiva ou provável, n (%)	Não reportado	18 (0,6)

ECAM: eventos cardiovasculares adversos maiores; IAM: infarto agudo do miocárdio; RLA: revascularização da lesão-alvo; RVA: revascularização do vaso-alvo; TS: trombose do stent.

## Discussão

Este estudo agrupou dados individuais de pacientes dos registros prospectivos INSPIRON e INSPIRON LATITUDE e representa até o momento a maior avaliação pós-comercialização desse SES com polímero biodegradável abluminal. A evolução da tecnologia dos *stents* coronários foi substancial, com os SF de nova geração demonstrando maior segurança e efetividade em comparação com dispositivos de gerações anteriores. Avanços no desenho dos *stents*, incluindo hastes mais finas, formulações poliméricas aprimoradas e agentes antiproliferativos mais eficazes, contribuíram para o perfil de segurança superior dos SF contemporâneos. Hastes mais finas não apenas melhoram a flexibilidade e a navegabilidade do dispositivo, mas também reduzem a lesão arterial e a inflamação induzidas pelo *stent*, ao mesmo tempo que facilitam a reendotelização.^6,16^ Revestimentos de polímeros duráveis têm sido associados à cicatrização arterial tardia e à inflamação persistente, conforme demonstrado em estudos de autópsia humana.^16,17^ Essas preocupações levaram ao desenvolvimento de SF com revestimentos de polímero biodegradável. Os SF com polímero biodegradável, particularmente aqueles com revestimento exclusivamente abluminal, podem favorecer cobertura mais rápida das hastes e cicatrização vascular em comparação com stents de polímero durável.^18^

O presente estudo avaliou o impacto do diâmetro do vaso coronário e do comprimento total do *stent* nos desfechos clínicos de longo prazo após ICP em uma população contemporânea de mundo real. Os principais achados podem ser resumidos da seguinte forma: i) o menor diâmetro do vaso coronário esteve associado a taxas significativamente mais elevadas de ECAM, mortalidade cardiovascular, nova revascularização e TS; ii) os pacientes com vasos menores apresentaram perfil clínico mais complexo, incluindo maior prevalência de DM e ICP prévia; iii) o comprimento total do *stent* esteve associado a ECAM nas análises não ajustadas, embora essa associação tenha deixado de ser significativa após o ajuste multivariado; e iv) apesar da inclusão de lesões coronárias complexas, os desfechos clínicos globais de longo prazo permaneceram favoráveis.

Um importante ponto forte desta análise é a inclusão de uma população ampla e não selecionada de pacientes consecutivos tratados na prática clínica habitual em vários centros brasileiros, incluindo uma proporção substancial de pacientes que se apresentaram com SCA e DAC anatomicamente complexa. Esses achados fornecem informações valiosas sobre o desempenho das plataformas contemporâneas de SF em contextos de assistência à saúde frequentemente sub-representados em ensaios clínicos randomizados.

A DAC de pequenos vasos continua sendo um dos cenários mais desafiadores da cardiologia intervencionista, apesar dos avanços substanciais na tecnologia dos *stents* e na terapia antiplaquetária.^19,20^ Historicamente, a ICP em pequenos vasos coronários tem sido associada a taxas mais elevadas de reestenose, RLA e ECAM do que a ICP em vasos maiores.^20^ No presente estudo, os pacientes com menor diâmetro dos vasos apresentaram taxas significativamente mais elevadas de ECAM durante o seguimento, particularmente em relação à mortalidade cardiovascular, RLA, RVA e TS definitiva ou provável. Diversos mecanismos podem explicar esses achados. Em primeiro lugar, vasos menores apresentam, inerentemente, menor área luminal final após o implante do *stent*. Consequentemente, mesmo uma proliferação neointimal modesta pode resultar em estreitamento luminal clinicamente significativo e isquemia recorrente. Além disso, artérias coronárias de pequeno calibre estão frequentemente associadas a aterosclerose difusa, lesões mais extensas, maior carga de calcificação e maior complexidade procedimental, fatores que podem contribuir para desfechos de longo prazo menos favoráveis.^19,21^

Além disso, a doença de pequenos vasos é comumente observada em pacientes com DM, condições inflamatórias crônicas e disfunção endotelial difusa.^22^ No presente estudo, os pacientes com menor diâmetro dos vasos apresentaram maior probabilidade de ter DM e dislipidemia, ambos preditores bem estabelecidos de reestenose e eventos isquêmicos recorrentes após ICP. O DM tem sido consistentemente associado ao aumento da proliferação neointimal, à progressão acelerada da aterosclerose e ao comprometimento da cicatrização vascular após o implante do *stent*. Consequentemente, os desfechos menos favoráveis observados após ICP em pequenos vasos podem refletir não apenas limitações anatômicas, mas também o substrato biológico adverso que frequentemente caracteriza essa população de pacientes.^22,23^

Outro achado importante do presente estudo foi a maior incidência de nova revascularização entre os pacientes com menor diâmetro dos vasos. As taxas de RLA e RVA foram significativamente mais elevadas nesse subgrupo, ressaltando o desafio persistente da reestenose em artérias coronárias de pequeno calibre, apesar dos avanços na tecnologia contemporânea dos SF. Embora os SF de geração atual tenham reduzido substancialmente as taxas de reestenose em comparação com os *stents* metálicos não farmacológicos e os SF de primeira geração, o impacto relativo da proliferação neointimal permanece maior em vasos menores devido às suas dimensões luminais basais limitadas.^19^

Curiosamente, apesar da associação significativa entre menor diâmetro do vaso e ECAM nas análises não ajustadas, o diâmetro médio do vaso não foi um preditor independente de ECAM após o ajuste por regressão de Cox multivariada. Esse achado sugere que o diâmetro do vaso pode não ser um determinante isolado do prognóstico, mas sim um marcador substituto da complexidade clínica e anatômica global. De fato, os pacientes com vasos menores apresentaram maior prevalência de diversas características clínicas de alto risco, incluindo DM, ICP prévia e DAC mais difusa. Após o ajuste para esses fatores, o impacto prognóstico do diâmetro do vaso foi atenuado.

No modelo multivariado final, idade mais avançada, doença do tronco da coronária esquerda ou doença arterial coronariana triarterial e SCA como indicação para ICP foram identificados como preditores independentes de ECAM. A associação entre idade avançada e piores desfechos cardiovasculares está bem estabelecida e provavelmente reflete maior carga de comorbidades, maior fragilidade e doença aterosclerótica mais avançada. Da mesma forma, a DAC de tronco da coronária esquerda e de múltiplos vasos constitui marcador estabelecido de maior carga isquêmica e complexidade anatômica e tem sido consistentemente associada a maior risco de ECAM em longo prazo.^24^

O impacto prognóstico do comprimento total do *stent* também foi avaliado. Segmentos tratados com maior comprimento total de *stent* estiveram associados a taxas mais elevadas de ECAM nas análises univariadas, incluindo maior incidência de eventos compostos durante o seguimento. Esse achado é biologicamente plausível, uma vez que lesões mais extensas geralmente refletem maior carga aterosclerótica, DAC mais difusa e maior complexidade procedimental. Além disso, um maior comprimento do *stent* tem sido historicamente associado a maiores taxas de reestenose, falha da lesão-alvo e trombose do *stent*.^25^

Entretanto, após o ajuste multivariado, o comprimento total do *stent* deixou de estar independentemente associado a ECAM. Essa observação pode refletir avanços importantes na tecnologia da ICP nos últimos anos.^26^ As plataformas contemporâneas de SF apresentam hastes mais finas, revestimentos poliméricos mais biocompatíveis e liberação mais eficiente de fármacos antiproliferativos, o que pode ter contribuído para reduzir os eventos adversos historicamente associados a segmentos coronários extensamente tratados com *stents*. Além disso, melhorias nas técnicas procedimentais, no preparo das lesões, nas estratégias de pós-dilatação e na farmacoterapia adjuvante podem ter atenuado ainda mais o impacto independente do comprimento do *stent* nos desfechos de longo prazo.^25,26^

Outro achado importante foi a progressão temporal dos ECAM durante o seguimento. A incidência cumulativa de ECAM aumentou entre 12 e 24 meses, indicando que o risco cardiovascular persiste além do período pós-procedimento inicial. Notavelmente, o acúmulo de eventos tardios pareceu ser impulsionado principalmente pela mortalidade e pela nova revascularização, e não apenas pelo IAM, sugerindo que a progressão da DAC subjacente e a complexidade das lesões podem contribuir substancialmente para o prognóstico de longo prazo após ICP.

Apesar da inclusão de pacientes com anatomia coronária complexa e características clínicas de alto risco, as taxas globais de ECAM permaneceram favoráveis durante todo o seguimento em comparação com aquelas relatadas em estudos clínicos anteriores.^27^ As taxas de TS e nova revascularização foram baixas, considerando a natureza de mundo real do registro. Notavelmente, a incidência de TS definitiva ou provável permaneceu abaixo de 1% aos 24 meses, apesar da inclusão de pacientes de alto risco e daqueles que se apresentaram com SCA. Esses achados reforçam ainda mais a segurança e a efetividade das estratégias contemporâneas de ICP e da tecnologia moderna dos SF na prática clínica habitual.^28,29^

### Limitações do estudo

Este estudo apresenta várias limitações. Primeiro, seu delineamento observacional está inerentemente sujeito a viés de seleção e confusão residual, apesar do ajuste multivariado. Segundo, os dados angiográficos não foram analisados por um laboratório central independente, o que pode ter introduzido variabilidade na avaliação das lesões e na mensuração dos vasos. Terceiro, modalidades de imagem intracoronária, como ultrassonografia intravascular e TCO, não foram utilizadas rotineiramente, limitando a avaliação detalhada da morfologia da placa, da expansão do *stent* e da otimização do procedimento. Quarto, o número relativamente pequeno de ECAM pode ter reduzido o poder estatístico das análises multivariadas. Por fim, como este estudo reflete a experiência de uma coorte de mundo real, comparações diretas com populações altamente selecionadas de ECR devem ser realizadas com cautela. Futuros ECR e estudos com seguimento mais prolongado são necessários para validar adicionalmente esses achados e avaliar os desfechos clínicos em longo prazo.

## Conclusões

Esta análise agrupada de dois grandes registros multicêntricos brasileiros demonstrou um perfil favorável de segurança e efetividade em longo prazo do SES Inspiron™ em uma população contemporânea e não selecionada de mundo real, incluindo pacientes submetidos a ICP de maior risco e com anatomia coronária complexa. O menor diâmetro do vaso esteve associado a desfechos clínicos não ajustados menos favoráveis, particularmente mortalidade cardiovascular, nova revascularização e TS, embora essas associações tenham sido atenuadas após o ajuste multivariado. De modo geral, as taxas de ECAM permaneceram baixas durante todo o seguimento, respaldando o uso do SES Inspiron™ na prática clínica contemporânea.

### Impacto na prática diária

Em uma grande coorte multicêntrica de mundo real, que incluiu uma proporção substancial de pacientes que se apresentaram com SCA e lesões coronárias complexas, o SES Inspiron™ demonstrou baixas taxas de IAM, nova revascularização e TS no seguimento de 2 anos, respaldando seu uso na prática clínica contemporânea.
