# Supplementary material for: Desfechos em 2 Anos do Stent Inspiron™ com Polímero Biodegradável e Eluição de Sirolimo em um Cenário de Mundo Real
Source: Arq Bras Cardiol. 2026 Nov 4;123(9):e20260244. [Article in Portuguese] doi: 10.36660/abc.20260244 (PMC13754393; doi:10.36660/abc.20260244)
Supplement: Translation

# Translation: Real-World 2-Year Outcomes of the Inspiron™ Biodegradable Polymer Sirolimus-Eluting Stent

**DOI:** 10.36660/abc.20260244i

**Authors:** Ricardo Czarnobai Soccol, Henrique Barbosa Ribeiro, Ricardo Lasevitch, Denise Pellegrini, Expedito Eustáquio Ribeiro da Silva, Carlos Alberto Santos de Mattos, Augusto Lima, Noriaki Takeshita, Sílvio Sérgio Pontes Câmara, José de Ribamar Costa, Leandro Zacarias Figueiredo de Freitas, Marcelo Sabedotti, Pablo Tomé Teixeirense, Márcio Augusto Meirelles Truffa, Ricardo de Gasperi, Ewerton de Souza Abreu, Ismael Arcari Bassani, José Armando Mangione, Maurício Cavalieri, Gustavo Martins Pereira Alves, Pedro Beraldo de Andrade, Rafael Maestri, Rafael Rebêlo César Cavalcanti, Maria Sanali Paiva, Marcelo de Oliveira Hirga, José Airton de Arruda, Flávio Correa Pivatelli, Julia Gonçalves Zandomenico, Paulo R. A. Caramori

**Affiliations:** 1 Brazil Hospital São Lucas da Pontifícia Universidade Católica do Rio Grande do Sul, Porto Alegre, RS – Brazil; 2 Brazil Instituto do Coração do Hospital das Clínicas da Faculdade de Medicina da Universidade de São Paulo, São Paulo, SP – Brazil; 3 Brazil Hospital de Clínicas de Passo Fundo, Passo Fundo, RS – Brazil; 4 Brazil Santa Casa de Misericórdia de Belo Horizonte, Belo Horizonte, MG – Brazil; 5 Brazil Hospital Angelina Caron, Campina Grande do Sul, PR – Brazil; 6 Brazil Hospital e Maternidade São Marcos, Teresina, PI – Brazil; 7 Brazil Instituto Dante Pazzanese de Cardiologia, São Paulo, SP – Brazil; 8 Brazil Santa Casa de Misericórdia de Goiânia, Goiânia, GO – Brazil; 9 Brazil Universidade de Caxias do Sul, Caxias do Sul, RS – Brazil; 10 Brazil Hospital dos Fornecedores de Cana de Piracicaba, Piracicaba, SP – Brazil; 11 Brazil Hospital Unimed Piracicaba, Piracicaba, SP – Brazil; 12 Brazil Hospital Paulo Sacramento, Jundiaí, SP – Brazil; 13 Brazil Associação Dr. Bartholomeu Tacchini (Hospital Tacchini), Bento Goncalves, RS – Brazil; 14 Brazil Hospital PRONTOCOR de Muriaé, Muriaé, MG – Brazil; 15 Brazil Hospital Beneficência Portuguesa de Pelotas, Pelotas, RS – Brazil; 16 Brazil Beneficência Portuguesa de São Paulo, São Paulo, SP – Brazil; 17 Brazil Hospital Alemão Oswaldo Cruz, São Paulo, SP – Brazil; 18 Brazil Hospital São Francisco de Assis, Belo Horizonte, MG – Brazil; 19 Brazil Samer Hospital, Resende, RJ – Brazil; 20 Brazil Santa Casa de Misericórdia de Marília, Marília, SP – Brazil; 21 Brazil Unimed Litoral, Balneário Camboriú, SC – Brazil; 22 Brazil Maestri e Kormann BC, Balneário Camboriú, SC – Brazil; 23 Brazil Hospital Memorial Arthur Ramos, Maceió, AL – Brazil; 24 Brazil Rede D’Or, Maceió, AL – Brazil; 25 Brazil Instituto Atena de Pesquisa Clínica, Maceió, AL – Brazil; 26 Brazil Hospital Policlin, São José dos Campos, SP – Brazil; 27 Brazil Hospital Meridional, Vitória, ES – Brazil; 28 Brazil Hospital de Base, São José do Rio Preto, SP – Brazil

**Keywords:** Biodegradable Polymers, Percutaneous Coronary Intervention, Stents, Sirolimus

## Abstract

**Background:**

The Inspiron™ sirolimus-eluting stent (SES) is a third-generation coronary stent featuring thin struts and an abluminal biodegradable polymer coating. Although it is widely used in Brazil, long-term real-world evidence remains limited.

**Objectives:**

To evaluate the safety and effectiveness of the Inspiron™ SES over 24 months in an unselected population undergoing percutaneous coronary intervention (PCI).

**Methods:**

This pooled analysis included 2,803 consecutive patients who underwent Inspiron™ SES implantation between June 2017 and January 2022 at 25 Brazilian centers. The primary endpoint was major adverse cardiovascular events (MACE), defined as the composite of cardiovascular death, MI, and target lesion revascularization (TLR) at 24 months. Kaplan-Meier survival analysis, Cox proportional hazards regression, and restricted cubic spline models were used. A two-sided p-value < 0.05 was considered statistically significant.

**Results:**

At 24 months, the cumulative incidence of MACE was 6.7%, with rates of 5.4% for all-cause mortality, 2.3% for cardiovascular mortality, 1.2% for MI, 0.9% for TLR, and 0.6% for definite or probable stent thrombosis (ST). Patients with a vessel diameter < 2.5 mm had significantly higher rates of MACE, cardiovascular death, repeat revascularization, and ST (p = 0.022). Independent predictors of MACE included older age (p = 0.034), left main or three-vessel coronary artery disease (p = 0.024), and acute coronary syndrome as the indication for PCI (p < 0.001).

**Conclusions:**

In this large real-world registry, the Inspiron™ SES demonstrated favorable safety and effectiveness through 24 months, with low rates of ST and repeat revascularization despite the inclusion of complex coronary lesions.

## Introduction

Percutaneous coronary intervention (PCI) is among the most commonly performed therapeutic procedures in contemporary cardiovascular practice.^1^ The introduction of drug-eluting stents (DES) markedly reduced in-stent restenosis by enabling the local delivery of antiproliferative agents to the arterial wall.^1,2^ However, first-generation DES were associated with increased thrombogenicity and a higher risk of late and very late stent thrombosis (ST).^3,4^ Permanent polymer coatings and thick-strut stent platforms have been implicated in delayed vascular healing, persistent inflammation, and increased thrombogenicity.^5,6^ These limitations drove the development of new-generation DES featuring thinner struts and biodegradable polymer coatings.^7,8^ When applied exclusively to the abluminal stent surface, biodegradable polymers may further promote vascular healing while reducing the risk of late major cardiovascular adverse events (MACE).^3^

The sirolimus-eluting stent (SES) Inspiron™ (Scitech Medical™, Aparecida de Goiânia, Brazil) was developed based on these principles. In the INSPIRON-I trial, the device demonstrated significantly lower late luminal loss and neointimal obstruction than its non–drug-eluting counterpart.^9^ In a randomized controlled trial (RCT) comparing the Inspiron™ with a biolimus-eluting stent, no significant angiographic differences were observed at 9 months, and clinical outcomes remained comparable through 5 years of follow-up.^10,11^ Intravascular imaging studies have also demonstrated favorable vascular healing and high rates of stent strut coverage following Inspiron™ implantation.^12,13^

Following Conformité Européenne (CE) Mark approval in 2020 and subsequent widespread clinical adoption, real-world evidence became essential to evaluate the device’s long-term safety and effectiveness. Notably, contemporary data in high-risk, anatomically complex all-comers populations treated in routine clinical practice remain limited. Therefore, this study aimed to evaluate the long-term real-world clinical outcomes associated with the SES Inspiron™.

## Methods

### Study design and population

The INSPIRON (ClinicalTrials.gov identifier: NCT03263260) and INSPIRON LATITUDE (ClinicalTrials.gov identifier: NCT03471234) were prospective multicenter registries conducted at 25 Brazilian centers. Given their similar study designs, patient-level data from both registries were pooled for the current analysis. Between June 2017 and January 2022, 2,803 consecutive patients who underwent PCI for native coronary artery lesion (CAL) with Inspiron™ stent implantation were enrolled.

Eligible patients were aged 18 years or older with angina symptoms and/or documented myocardial ischemia who underwent treatment of a native CAL with the SES. Angiographic eligibility criteria included lesion length < 34 mm and a reference vessel diameter of 2.5-4.0 mm. Exclusion criteria comprised target lesions located in saphenous vein or arterial grafts and treatment with any stent other than the study device during the index procedure.

All procedures were performed according to standard clinical practice. The choice of vascular access, stent size, and interventional strategy was left to the discretion of the treating operator. Mean vessel diameter was defined as the average reference vessel diameter of the treated lesions during the index procedure and was determined according to the operator’s angiographic assessment during PCI. Quantitative coronary angiography was encouraged whenever feasible; however, visual angiographic estimation was used in most cases, reflecting routine real-world practice. Intracoronary imaging guidance was not mandated by the study protocol. Dual antiplatelet therapy with aspirin and a P2Y12 inhibitor was recommended for at least 6 months after stent implantation.

The study was conducted in accordance with the Declaration of Helsinki and was approved by the human research ethics committee at each participating center. All patients provided written informed consent before enrollment.

### Stent design

The study device is a thin-strut (75 μm) cobalt-chromium SES with an abluminal biodegradable polymer coating composed of poly-L-lactic acid and poly(lactic-co-glycolic acid). It elutes sirolimus at a low dose (1.4 µg/mm^2^), resulting in a moderate drug-release profile, with 60% of the drug released within 10 days and complete release by 45 days. Complete polymer degradation occurs between 6 and 9 months (data on file, Scitech Medical™, Brazil). The REPAIR trial evaluated the vascular healing profile of the Inspiron™ stent using serial optical coherence tomography (OCT) and demonstrated a high rate of strut coverage, with 97% of struts covered at 3 months.^13^

### Study endpoints

The primary endpoint was the incidence of MACE, defined as the composite of cardiovascular death, myocardial infarction (MI), and target lesion revascularization (TLR) at 24 months. Secondary endpoints included all-cause mortality, cardiovascular mortality, any MI, TLR, target vessel revascularization (TVR), and definite or probable ST at 24 months.

### Definitions

Device success was defined as successful delivery and deployment of the study stent with residual diameter stenosis < 30%, as assessed by quantitative coronary angiography or visual estimation. Procedural success was defined as device success without in-hospital death, ST-segment elevation MI, acute or subacute ST, or the need for emergency TVR.

MI was defined according to the contemporaneous Third Universal Definition.^14^ In accordance with the Academic Research Consortium criteria,^15^ all deaths were classified as cardiac unless an unequivocal noncardiac cause was established. Definite ST was defined as angiographically confirmed thrombosis with Thrombolysis in Myocardial Infarction flow grade 0 or 1, or the presence of a flow-limiting thrombus associated with acute ischemic symptoms, regardless of any intervening revascularization procedure. ST was classified according to timing as acute (0-24 hours), subacute (> 24 hours to 30 days), late (30 days to 1 year), or very late (> 1 year). Probable ST was defined as unexplained death within 30 days or target vessel MI without angiographic confirmation of thrombosis or identification of another culprit lesion. Possible ST was defined as unexplained death occurring after 30 days.

TLR was defined as any repeat PCI of the target lesion performed for restenosis. The target lesion comprised the treated segment extending 5 mm proximal and 5 mm distal to the stent edges. TVR was defined as any repeat PCI involving any segment of the target vessel.

### Clinical follow-up

Baseline clinical, angiographic, and procedural data were prospectively collected in a dedicated database at each participating center. All patients were followed through hospital discharge. Clinical follow-up was scheduled at 30 days, 12 months, and 24 months after the index procedure and was conducted through outpatient visits or structured telephone interviews according to local institutional practice. Additional information was obtained through review of medical records and direct communication with referring physicians whenever necessary. Clinical events were reported by each participating center according to prespecified protocol definitions. The study coordinating center performed additional review of reported clinical events and follow-up data as needed to ensure the completeness, accuracy, and consistency of clinical event adjudication across participating centers.

### Statistical analysis

Statistical analyses were performed using the pooled database from the INSPIRON and INSPIRON LATITUDE registries, comprising 2,803 patients who underwent PCI. Patients were categorized according to tertiles of mean vessel diameter (< 2.5 mm, 2.5-3.0 mm, and > 3.0 mm) and total stent length implanted during the index procedure (< 25 mm, 25-30 mm, and > 30 mm).

The primary endpoint was MACE, defined as the composite of cardiovascular death, MI, and TLR. Secondary endpoints included i) the composite of all-cause mortality, MI, and TLR; ii) all-cause mortality; iii) cardiovascular mortality; iv) any MI; v) target vessel MI; vi) TLR; vii) TVR; and viii) definite or probable ST.

Continuous variables were normally distributed and are presented as mean ± SD. Comparisons among the three groups were performed using one-way analysis of variance followed by Tukey’s post hoc test for pairwise comparisons when appropriate. Categorical variables were compared using the chi-square test.

Event-free survival was estimated using the Kaplan-Meier method and compared with the log-rank test. Univariable and multivariable Cox proportional hazards regression models were used to evaluate the associations of mean vessel diameter and total stent length with the occurrence of MACE during the 24-month follow-up. The proportional hazards assumption was assessed using Schoenfeld residuals, and no significant violations were detected.

To evaluate potential nonlinear associations between mean vessel diameter or total stent length and the probability of MACE, restricted cubic spline regression models with five knots were constructed using the *rcspline*.*plot* function from the *Hmisc* package in R. Both parametric and nonparametric estimates of the logistic regression function were generated using Friedman’s SuperSmoother (*supsmu*) smoothing algorithm.

All statistical tests were two-sided, and a p-value < 0.05 was considered statistically significant. Statistical analyses were performed using R version 3.5.3 (R Foundation for Statistical Computing) and RStudio version 1.1.4.

## Results

A total of 2,803 patients were included in the current analysis. Baseline clinical characteristics according to mean vessel diameter are summarized in Table 1. Patients with smaller vessel diameters were older and more likely to be female. Diabetes mellitus (DM), including both insulin-dependent and non–insulin-dependent disease, was more prevalent among patients with a vessel diameter < 2.5 mm. Dyslipidemia and prior PCI were also more common in this group. Conversely, current smoking was more frequent among patients with a vessel diameter > 3.0 mm. Notably, more than half of the study population presented with acute coronary syndrome (ACS), underscoring the real-world, high-risk nature of the cohort.

**Table 1:** – Baseline clinical characteristics according to mean vessel diameter

Variable	Overall (n = 2,803)	Q1: < 2.5 mm (n = 688)	Q2: 2.5-3.0 mm (n = 1,263)	Q3: > 3.0 mm (n = 852)	p-value
Age, years	62.89 ± 10.81	64.14 ± 10.58	62.81 ± 10.56	62.00 ± 11.27	0.001
Male sex, n (%)	1,812 (64.6)	383 (55.7)	811 (64.2)	618 (72.5)	< 0.001
Hypertension, n (%)	2,133 (76.1)	533 (77.5)	971 (76.9)	629 (73.8)	0.169
DM, n (%)	1,024 (36.5)	312 (45.3)	472 (37.4)	240 (28.2)	< 0.001
Dyslipidemia, n (%)	1,459 (52.1)	390 (56.7)	645 (51.1)	424 (49.8)	0.017
Former smoking, n (%)	645 (23.0)	148 (21.5)	280 (22.2)	217 (25.5)	0.117
Current smoking, n (%)	557 (19.9)	112 (16.3)	243 (19.2)	202 (23.7)	0.001
Prior MI, n (%)	16 (0.6)	4 (0.6)	8 (0.6)	4 (0.5)	0.869
CKD, n (%)	97 (3.5)	19 (2.8)	48 (3.8)	30 (3.5)	0.484
Prior CABG, n (%)	112 (4.0)	33 (4.8)	50 (4.0)	29 (3.4)	0.380
Prior PCI, n (%)	493 (17.6)	144 (20.9)	207 (16.4)	142 (16.7)	0.029
Clinical presentation					0.011
Stable angina, n (%)	1,369 (48.8)	367 (53.5)	585 (49.4)	379 (44.8)	
ACS, n (%)	1,434 (51.1)	321 (46.5)	640 (50.6)	473 (55.4)	

ACS: acute coronary syndrome; CABG: coronary artery bypass grafting; CKD: chronic kidney disease; DM: diabetes mellitus; MI: myocardial infarction; PCI: percutaneous coronary intervention.

Baseline characteristics according to total stent length are presented in Table 2. Patients treated with longer stents were more likely to be male and to present with ACS at admission. Current smoking was also more prevalent among patients receiving longer stents.

**Table 2:** – Angiographic and procedural characteristics according to total stent length

Variable	Overall (n = 2,803)	< 25 mm (n = 1,944)	25-30 mm (n = 419)	> 30 mm (n = 440)	p-value
**Target coronary vessel**					
LAD, n (%)	1,187 (42.3)	842 (43.3)	176 (42.0)	169 (38.4)	0.178
LCx, n (%)	512 (18.3)	384 (19.8)	63 (15.0)	65 (14.8)	0.018
RCA, n (%)	1,038 (37.0)	685 (35.2)	169 (40.3)	184 (41.8)	0.021
Left main, n (%)	34 (1.2)	24 (1.2)	1 (0.2)	9 (2.0)	0.053
**Lesion type**					
De novo lesion, n (%)	2,638 (94.1)	1,834 (94.3)	391 (93.3)	413 (93.9)	0.721
In-stent restenosis, n (%)	165 (5.9)	110 (5.7)	28 (6.7)	27 (6.1)	0.721
Mean vessel diameter, mm	3.00 ± 0.45	2.98 ± 0.47	3.00 ± 0.44	3.04 ± 0.35	0.038
Mean lesion length, mm	21.02 ± 10.41	16.24 ± 5.57	27.44 ± 7.36	35.98 ± 11.72	< 0.001
Predilation stenosis, %	85.93 ± 10.20	85.40 ± 10.06	86.51 ± 10.41	87.75 ± 10.39	< 0.001
Postdilation stenosis, %	1.75 ± 4.36	1.62 ± 4.12	1.83 ± 4.51	2.21 ± 5.03	0.084
Number of stents implanted per PCI	1.38 ± 0.96	1.37 ± 0.96	1.51 ± 1.04	1.32 ± 0.90	0.008
Mean stent length, mm	22.81 ± 8.18	18.33 ± 3.57	28.36 ± 1.17	37.28 ± 6.42	< 0.001
Mean stent diameter, mm	3.00 ± 0.45	2.98 ± 0.47	3.00 ± 0.44	3.04 ± 0.35	0.038

LAD: left anterior descending artery; LCx: left circumflex artery; PCI: percutaneous coronary intervention; RCA: right coronary artery.

Lesion length increased progressively with increasing vessel diameter. Patients with smaller vessels required a greater number of stents despite having shorter mean stent lengths. Mean stent diameter increased proportionally across vessel diameter categories. Procedural success rates were uniformly high (> 99%) and did not differ significantly among groups.

When patients were stratified according to total stent length, lesion complexity increased progressively across categories. Patients receiving longer stents had significantly longer lesions and greater baseline stenosis severity.

At 24 months, the incidence of the primary endpoint, MACE, differed significantly across vessel diameter groups (p = 0.022). Patients with a vessel diameter < 2.5 mm had the highest incidence of MACE (5.4%), whereas those with a vessel diameter > 3.0 mm had the lowest incidence (2.7%). Cardiovascular mortality also differed significantly among groups, occurring more frequently in patients with smaller vessels (3.6% vs. 2.1% vs. 1.4%; p = 0.013).

Although crude cumulative event rates at 24 months differed significantly across vessel diameter groups, Kaplan-Meier analysis did not demonstrate significant differences in event-free survival by the log-rank test (p = 0.37). This discrepancy may reflect differences in event timing, censoring patterns, and the relatively small number of MACE during follow-up. Kaplan-Meier analysis showed an early increase in MACE among patients with a vessel diameter < 2.5 mm during the first 60 days after PCI, followed by another increase after approximately 16 months of follow-up (Figure 1A). Patients with intermediate vessel diameters (2.5-3.0 mm) exhibited a delayed increase in event rates beginning at approximately 12 months.

**Figure 1:** – Kaplan-Meier curves for MACE at 24-month follow-up. A) Stratified by mean vessel diameter. B) Stratified by total stent length (sum of the lengths of all stents implanted during percutaneous coronary intervention). MACE: major adverse cardiovascular events.

Rates of TLR and ST were also significantly higher among patients with smaller vessel diameters. In contrast, no significant differences were observed in all-cause mortality or MI.

Restricted cubic spline analysis demonstrated a significant overall association between mean vessel diameter and the probability of MACE, which suggests a nonlinear relationship between vessel caliber and MACE (Figure 2). The spline curve indicated a progressive decline in the estimated risk of MACE with increasing vessel diameter.

**Figure 2:** – Restricted cubic spline regression of the probability of MACE (cardiovascular death, myocardial infarction, or target lesion revascularization) according to mean vessel diameter. AIC: Akaike information criterion; LR: likelihood ratio; MACE: major adverse cardiovascular events.

In univariable Cox regression analysis, mean vessel diameter was significantly associated with MACE (p = 0.022). However, after multivariable adjustment using stepwise Cox regression, mean vessel diameter was no longer an independent predictor of MACE. The final multivariable model identified older age (p = 0.034), left main or three-vessel coronary artery disease (CAD) (p = 0.024), and ACS as the indication for PCI (p < 0.001) as independent predictors of MACE, with a concordance index of 0.71. These findings are presented in Figure 3.

**Figure 3:** – Stepwise multivariable Cox regression model for major adverse cardiovascular events (cardiovascular death, myocardial infarction, and target lesion revascularization) during 24-month follow-up. ACS: acute coronary syndrome; AIC: Akaike information criterion; CABG: coronary artery bypass grafting; CAD: coronary artery disease; CKD: chronic kidney disease; PCI: percutaneous coronary intervention.

The incidence of MACE increased numerically with increasing total stent length, from 3.5% among patients treated with stents < 25 mm to 5.7% among those receiving stents > 30 mm; however, this difference did not reach statistical significance (p = 0.070).

Similarly, Kaplan-Meier curves demonstrated overlapping cumulative incidences of MACE across stent length categories throughout the 24-month follow-up, with no significant differences by the log-rank test (p = 0.51) (Figure 1B).

Nevertheless, longer total stent length was significantly associated with the secondary composite endpoint of all-cause mortality, MI, or TLR (p = 0.018). This finding appeared to be driven primarily by a numerically higher rate of all-cause mortality among patients receiving stents > 30 mm at 24 months (7.3%; p = 0.100).

Restricted cubic spline analysis suggested a gradual increase in the estimated probability of MACE with increasing total stent length, although without evidence of a strong linear association (Figure 4). In multivariable Cox regression analysis, total stent length was not independently associated with MACE (p = 0.264).

**Figure 4:** – Restricted cubic spline regression of the probability of MACE (cardiovascular death, myocardial infarction, or target lesion revascularization) according to total stent length (sum of the lengths of all stents implanted during percutaneous coronary intervention). AIC: Akaike information criterion; LR: likelihood ratio; MACE: major adverse cardiovascular events.

Patients who developed MACE during follow-up were more likely to present with ACS, kidney disease, prior coronary artery bypass grafting, prior PCI, and a history of smoking. They also had significantly greater total stent length and smaller mean stent diameter than patients who did not develop MACE (Table 3).

**Table 3:** – Clinical and procedural characteristics according to the occurrence of MACE during 24-month follow-up

Variable	No MACE (n = 2,697)	MACE (n = 106)	p-value
Age, years	62.74 ± 10.79	66.12 ± 10.85	0.002
Male sex, n (%)	1,748 (64.8)	64 (60.4)	0.366
ACS, n (%)	1,367 (50.7)	67 (63.2)	0.014
Hypertension, n (%)	2,048 (75.9)	85 (80.2)	0.318
DM, n (%)	973 (36.1)	51 (48.1)	0.015
Dyslipidemia, n (%)	1,397 (51.8)	62 (58.5)	0.182
Current smoking, n (%)	530 (19.7)	27 (25.5)	0.159
Former smoking, n (%)	604 (22.4)	41 (38.7)	< 0.001
CKD, n (%)	86 (3.2)	11 (10.4)	< 0.001
Prior MI, n (%)	14 (0.5)	2 (1.9)	0.082
Prior CABG, n (%)	101 (3.7)	11 (10.4)	0.001
Prior PCI, n (%)	456 (16.9)	37 (34.9)	< 0.001
Left main or three-vessel CAD, n (%)	278 (10.3)	23 (21.7)	< 0.001
Mean vessel diameter, mm	3.01 ± 0.45	2.82 ± 0.41	< 0.001
Mean lesion length, mm	20.79 ± 10.28	26.11 ± 12.48	< 0.001
Total stent length, mm	30.92 ± 24.91	47.30 ± 36.42	< 0.001
Number of stents implanted	1.36 ± 0.94	1.74 ± 1.18	< 0.001
Mean stent diameter, mm	3.01 ± 0.45	2.78 ± 0.39	< 0.001
Mean stent length, mm	22.63 ± 8.07	27.42 ± 9.84	< 0.001
Angiographic success, n (%)	2,498 (99.8)	99 (99.0)	0.089

ACS: acute coronary syndrome; CABG: coronary artery bypass grafting; CAD: coronary artery disease; CKD: chronic kidney disease; DM: diabetes mellitus; MACE: major adverse cardiovascular events; MI: myocardial infarction; PCI: percutaneous coronary intervention.

Overall, these findings suggest that smaller coronary vessel diameter is associated with less favorable cardiovascular outcomes after PCI, particularly with respect to cardiovascular mortality and repeat revascularization, whereas total stent length appears to be a marker of lesion complexity rather than an independent predictor of MACE after multivariable adjustment.

Selected clinical outcomes at 12 and 24 months are presented in Table 4. The cumulative incidence of MACE increased progressively over time, accompanied by gradual increases in all-cause mortality, cardiovascular mortality, MI, repeat revascularization, and ST. Overall, rates of MACE remained low throughout the study period. These findings are also summarized in the Central Illustration.

**Table 4:** – Clinical outcomes at 12- and 24-month follow-up

Endpoint	12 months (n = 2,803)	24 months (n = 2,803)
MACE, n (%)	106 (3.8)	189 (6.7)
All-cause mortality, n (%)	101 (3.6)	151 (5.4)
Cardiovascular mortality, n (%)	46 (1.6)	64 (2.3)
MI, n (%)	27 (1.0)	35 (1.2)
TVR, n (%)	Not reported	33 (1.2)
TLR, n (%)	Not reported	26 (0.9)
Definite or probable ST, n (%)	Not reported	18 (0.6)

MACE: major adverse cardiovascular events; MI: myocardial infarction; ST: stent thrombosis; TLR: target lesion revascularization; TVR: target vessel revascularization.

## Discussion

This study pooled patient-level data from the prospective INSPIRON and INSPIRON LATITUDE registries and represents the largest postmarketing evaluation to date of this abluminal biodegradable polymer SES. The evolution of coronary stent technology has been substantial, with new-generation DES demonstrating improved safety and effectiveness compared with earlier-generation devices. Advances in stent design, including thinner struts, improved polymer formulations, and more effective antiproliferative agents, have contributed to the superior safety profile of contemporary DES. Thinner struts not only improve device flexibility and deliverability but also reduce stent-induced arterial injury and inflammation while facilitating re-endothelialization.^6,16^ Durable polymer coatings have been associated with delayed arterial healing and persistent inflammation, as demonstrated in human autopsy studies.^16,17^ These concerns led to the development of DES with biodegradable polymer coatings. Biodegradable polymer DES, particularly those with abluminal-only coating, promote more rapid vascular healing and re-endothelialization.^18^

The current study evaluated the impact of coronary vessel diameter and total stent length on long-term clinical outcomes after PCI in a contemporary real-world population. The main findings can be summarized as follows: i) smaller coronary vessel diameter was associated with significantly higher rates of MACE, cardiovascular mortality, repeat revascularization, and ST; ii) patients with smaller vessels had a more complex clinical profile, including a higher prevalence of DM and prior PCI; iii) total stent length was associated with MACE in unadjusted analyses, although this association was no longer significant after multivariable adjustment; and iv) despite the inclusion of complex coronary lesions, overall long-term clinical outcomes remained favorable.

An important strength of this analysis is the inclusion of a large, unselected all-comers population treated in routine clinical practice across multiple Brazilian centers, including a substantial proportion of patients presenting with ACS and anatomically complex CAD. These findings provide valuable insights into the performance of contemporary DES platforms in health care settings that are often underrepresented in RCTs.

Small-vessel CAD remains one of the most challenging scenarios in interventional cardiology despite substantial advances in stent technology and antiplatelet therapy.^19,20^ Historically, PCI in small coronary vessels has been associated with higher rates of restenosis, TLR, and MACE than PCI in larger vessels.^20^ In the current study, patients with smaller vessel diameters experienced significantly higher rates of MACE during follow-up, particularly with respect to cardiovascular mortality, TLR, TVR, and definite or probable ST. Several mechanisms may explain these findings. First, smaller vessels inherently achieve a smaller final luminal area following stent implantation. Consequently, even modest neointimal proliferation may result in clinically significant luminal narrowing and recurrent ischemia. In addition, small coronary arteries are frequently associated with diffuse atherosclerosis, longer lesions, greater calcific burden, and increased procedural complexity, all of which may contribute to less favorable long-term outcomes.^19,21^

Moreover, small-vessel disease is commonly observed in patients with DM, chronic inflammatory conditions, and diffuse endothelial dysfunction.^22^ In the current study, patients with smaller vessel diameters were more likely to have DM and dyslipidemia, both well-established predictors of restenosis and recurrent ischemic events after PCI. DM has consistently been associated with increased neointimal proliferation, accelerated progression of atherosclerosis, and impaired vascular healing following stent implantation. Consequently, the less favorable outcomes observed after PCI in small vessels may reflect not only anatomical constraints but also the adverse biological substrate that frequently characterizes this patient population.^22,23^

Another important finding of the current study was the higher incidence of repeat revascularization among patients with smaller vessel diameters. Rates of both TLR and TVR were significantly higher in this subgroup, underscoring the persistent challenge of restenosis in small coronary arteries despite advances in contemporary DES technology. Although current-generation DES have substantially reduced restenosis rates compared with bare-metal stents and first-generation DES, the relative impact of neointimal proliferation remains greater in smaller vessels because of their limited baseline luminal dimensions.^19^

Interestingly, despite the significant association between smaller vessel diameter and MACE in unadjusted analyses, mean vessel diameter was not an independent predictor of MACE after multivariable Cox regression adjustment. This finding suggests that vessel diameter may not be an isolated determinant of prognosis but rather a surrogate marker of overall clinical and anatomical complexity. Indeed, patients with smaller vessels had a higher prevalence of several high-risk clinical characteristics, including DM, prior PCI, and more diffuse CAD. After adjustment for these factors, the prognostic impact of vessel diameter was attenuated.

In the final multivariable model, older age, left main or three-vessel CAD, and ACS as the indication for PCI emerged as independent predictors of MACE. The association between advanced age and worse cardiovascular outcomes is well established and likely reflects a greater burden of comorbidities, increased frailty, and more advanced atherosclerotic disease. Likewise, left main and multivessel CAD are established markers of greater ischemic burden and anatomical complexity and have consistently been associated with an increased risk of long-term MACE.^24^

The prognostic impact of total stent length was also evaluated. Longer stented segments were associated with higher rates of MACE in univariable analyses, including a higher incidence of composite events during follow-up. This finding is biologically plausible, as longer lesions generally reflect a greater atherosclerotic burden, more diffuse CAD, and increased procedural complexity. In addition, greater stent length has historically been associated with higher rates of restenosis, target lesion failure, and ST.^25^

However, after multivariable adjustment, total stent length was no longer independently associated with MACE. This observation may reflect important advances in PCI technology in recent years.^26^ Contemporary DES platforms feature thinner struts, more biocompatible polymer coatings, and more efficient antiproliferative drug delivery, substantially reducing the MACE historically associated with extensive stented segments. Furthermore, improvements in procedural techniques, lesion preparation, postdilation strategies, and adjunctive pharmacotherapy may have further mitigated the independent impact of stent length on long-term outcomes.^25, 26^

Another important finding was the temporal progression of MACE during follow-up. The cumulative incidence of MACE increased between 12 and 24 months, indicating that cardiovascular risk persists beyond the early postprocedural period. Notably, the accumulation of late events appeared to be driven primarily by mortality and repeat revascularization rather than MI alone, suggesting that progression of underlying CAD and lesion complexity may contribute substantially to long-term prognosis after PCI.

Despite the inclusion of patients with complex coronary anatomy and high-risk clinical characteristics, overall rates of MACE remained favorable throughout follow-up compared with those reported in previous clinical studies.^27^ Rates of ST and repeat revascularization were low considering the real-world nature of the registry. Notably, the incidence of definite or probable ST remained below 1% at 24 months, despite the inclusion of high-risk patients and those presenting with ACS. These findings further support the safety and effectiveness of contemporary PCI strategies and modern DES technology in routine clinical practice.^28,29^

### Study limitations

This study has several limitations. First, its observational design is inherently subject to selection bias and residual confounding despite multivariable adjustment. Second, angiographic data were not analyzed by an independent core laboratory, potentially introducing variability in lesion assessment and vessel sizing. Third, intracoronary imaging modalities, such as intravascular ultrasound and OCT, were not routinely used, limiting detailed assessment of plaque morphology, stent expansion, and procedural optimization. Fourth, the relatively small number of MACE may have reduced the statistical power of the multivariable analyses. Finally, because this study reflects the experience of a real-world cohort, direct comparisons with highly selected RCT populations should be made with caution. Future RCTs and studies with longer follow-up are warranted to further validate these findings and evaluate long-term clinical outcomes.

## Conclusions

This pooled analysis of two large multicenter Brazilian registries demonstrated favorable long-term safety and effectiveness of the Inspiron™ SES in a contemporary real-world all-comers population, including patients undergoing high-risk and anatomically complex PCI. Smaller vessel diameter was associated with less favorable unadjusted clinical outcomes, particularly cardiovascular mortality, repeat revascularization, and ST, although these associations were attenuated after multivariable adjustment. Overall, rates of MACE remained low throughout follow-up, supporting the use of the Inspiron™ SES in routine contemporary PCI practice.

### Impact on daily practice

In a large multicenter real-world cohort that included a substantial proportion of patients presenting with ACS and complex coronary lesions, the Inspiron™ SES demonstrated low rates of MI, repeat revascularization, and ST at 2-year follow-up, supporting its use in routine contemporary PCI practice.
